# 长春瑞滨联合奥沙利铂对比长春瑞滨联合顺铂治疗中晚期非小细胞肺癌的系统评价

**DOI:** 10.3779/j.issn.1009-3419.2010.02.06

**Published:** 2010-02-20

**Authors:** 夏 刘, 力 马, 克虎 杨, 金徽 田

**Affiliations:** 1 300052 天津, 天津医科大学总医院, 天津市胸部肿瘤中心肿瘤内科 Department of Medical Oncology, Tianjin Thoracic Cancer Center, Tianjin Medical University General Hospital, Tianjin 300052, China; 2 730000 兰州, 兰州大学循证医学中心, 兰州大学基础医学院 Evidence-based Medicine Center of Lanzhou University, School of Basic Medical Science of Lanzhou University, Lanzhou 730000, China

**Keywords:** 肺肿瘤, 长春瑞滨, 奥沙利铂, 顺铂, 系统评价, Lung neoplasms, Vinorelbine, Oxaliplatin, Cisplatin, Systematic review

## Abstract

**背景与目的:**

顺铂联合长春瑞滨是目前治疗中晚期非小细胞肺癌的首选方案之一, 奥沙利铂对非小细胞肺癌同样有效且胃肠道、肾毒性、骨髓抑制较轻。本研究旨在系统评价长春瑞滨联合奥沙利铂方案(NO)对比长春瑞滨联合顺铂方案(NP)治疗中晚期非小细胞肺癌的疗效和毒副作用的差异。

**方法:**

计算机检索CBM、CNKI、VIP、Cochrane Library、PubMed、EMBASE等数据库及美国临床肿瘤学会(ASCO)论文集, 并辅以手工检索和其它检索, 对纳入文献进行方法学质量评价, 采用RevMan 5.0软件进行*meta*分析。

**结果:**

共纳入14个研究(1 270例患者)。NO和NP方案在客观缓解率、疾病控制率、1年生存率、贫血、血小板减少发生率方面的差异无统计学意义; NP方案的消化道毒性、白细胞减少、脱发、肾毒性较NO方案明显(*P* < 0.05), NO方案神经毒性高于NP方案(*P* < 0.05)。

**结论:**

NO方案和NP方案治疗中晚期非小细胞肺癌疗效相似, 副作用方面有差异, NO可能更易耐受。

## Abstract (en)

**Background and objective:**

Cisplatin (DDP) plus vinorelbine (NVB) constitute the first-line regimen (NP regimen) for non-small cell lung cancer (NSCLC).Oxaliplatin (OXA) is another effective drug in treatment of NSCLC with mild toxicities to gastrointestinal tract, kidney and bone marrow.The aim of this study is to evaluate the efficiency and safety between NVB plus OXA (NO) regimen and NP regimen for advanced NSCLC.

**Methods:**

We searched CBM, CNKI, VIP, Cochrane Library, PubMed, EMBASE, ASCO *etc*.conference proceedings and internet information.Randomized controlled trials of NO versus NP for advanced NSCLC were included; we evaluated the quality of the included studies and analyzed data by Cochrane Collaboration's RevMan 5.0 software.

**Results:**

Fourteen randomized trials involving 1 270 patients were included.There were no statistical differences between NO and NP in overall response rate, disease control rate, 1-year survival rate, anemia and thrombocytopenia.Gastrointestinal toxicity, leucopenia, alopecia and kidney toxicity were more serious in NP (*P* < 0.05), but neuritis was more serious in NO, with significant difference (*P* < 0.05).

**Conclusion:**

The clinical efficacy of NO and NP for advanced NSCLC was similar, but the side effects were different.The toxicity of NO has the tendency to be more tolerable.

肺癌是一种恶性程度极高且易复发、转移的恶性肿瘤, 在新诊断的肺癌中, 非小细胞肺癌(non-small cell lung cancer, NSCLC)占80%-85%, 而其中75%为不能手术的中晚期NSCLC且预后极差, 1年生存率为30%-35%, 中位生存期仅为8个月-10个月^[1]^。

几十年来, 以铂类为基础的化疗在NSCLC治疗中占主导地位, 其中第一代铂类顺铂(DDP)疗效肯定, 是国家综合癌症网(NCCN)指南推荐的一线基础用药。铂类联合新一代化疗药物是目前治疗中晚期NSCLC的首选方案^[2]^。长春瑞滨(NVB)是治疗NCSLC最有效的新药之一, 单药有效率为12.5%-30%^[3]^, 与顺铂具有协同作用, 长春瑞滨联合顺铂(NP)方案成为治疗NSCLC最常见且疗效确切的方案。国内报道NP方案治疗中晚期NSCLC有效率为32%-48%^[4-17]^, 但顺铂的消化道毒性、肾脏毒性和耳毒性明显, 很多老年患者及身体状况较差者难以耐受, 促使新的铂类药物不断问世。奥沙利铂(OXA)为第三代铂类新药, 几乎不存在肾脏、耳毒性, 消化道毒性及血液学毒性轻微, 虽有外周神经感觉异常, 但症状轻微且可逆, 已广泛应用于NSCLC的治疗。奥沙利铂与长春瑞滨联合(NO)方案治疗中晚期NSCLC总有效率达50%左右^[18, 19]^。

NO作为新的联合化疗方案, 是否比传统NP方案具有更好的疗效及安全性, 能否取代NP成为中晚期NSCLC的首选治疗方案, 目前尚无定论。本研究参照Cochrane系统评价的方法, 对所有关于NO方案和NP方案治疗中晚期NSCLC的疗效和副作用进行评价, 为临床决策提供依据。

## 1 材料与方法

### 1.1 纳入标准

#### 1.1.1 研究类型

随机对照试验, 无论是否采用盲法。

#### 1.1.2 研究对象

经病理组织学或细胞学确诊的Ⅲ、Ⅳ期NSCLC患者, 且ZPS(Zubrod-ECOG-WHO)评分≤2分, 血常规、心、肝、肾功能正常, 预计生存期 > 3个月。

#### 1.1.3 干预措施

长春瑞滨联合顺铂(NP)*vs*长春瑞滨联合奥沙利铂(NO)。

#### 1.1.4 观察指标

客观缓解率(完全缓解+部分缓解); 疾病控制率(完全缓解+部分缓解+稳定); 1年生存率; 消化道毒性、血液学毒性、脱发、神经毒性、肾脏毒性等。

### 1.2 检索策略

以“(非小细胞肺癌OR非小细胞肺肿瘤)AND(奥沙利铂OR草酸铂OR乐沙定OR艾恒)AND(长春瑞滨OR诺维本OR盖诺)AND顺铂”检索中国期刊全文数据库(1994-2009.9)、中国生物医学文献数据库(1978-2009.9)、中文科技期刊全文数据库(1989-2009.9);以“(non-small cell lung carcinoma OR non-small cell lung neoplasms OR non-small cell lung cancer)AND oxaliplatin AND vinorelbine AND cisplatin”检索PubMed(1996-2009.9)、Cochrane library(2009年第3期)、EMBASE(1974-2009.9)、美国临床肿瘤学会(ASCO)论文集(1995-2009)。手工检索相关中文期刊, 并用Google Scholar、Medical Martix等搜索引擎在互联网上查找相关文献, 追查已纳入文献的参考文献, 与本领域专家、通讯作者等联系以获取以上检索未发现的相关信息。如试验报告不详或资料缺乏, 通过信件与作者联系获取。

### 1.3 文献筛选和资料提取

两位研究者独立阅读所获文献题目和摘要, 在排除明显不符合纳入标准的试验后, 对可能符合纳入标准的试验阅读全文, 以确定是否符合纳入标准。两位评价者交叉核对纳入试验的结果, 对有分歧而难以确定是否纳入的试验通过讨论或由第三位评价者决定其是否纳入。提取资料主要包括:①一般资料:题目、作者姓名、发表日期和文献来源; ②研究特征:研究对象的一般情况、各组病人的基线可比性、干预措施; ③结局指标:客观缓解率、疾病控制率、1年生存率, 治疗引起的并发症包括消化道毒性、血液学毒性、脱发、神经毒性、肾脏毒性。

### 1.4 质量评价

纳入文献的方法学质量依据Cochrane评价手册4.2.6随机对照试验质量的4条质量评价标准进行评价:①采用何种随机分配方法, 方法是否正确; ②是否进行分配隐藏, 方法是否正确; ③是否采用盲法, 对哪些人实施了盲法; ④有无失访和退出, 是否采用意向性分析(intention to treat, ITT)。

### 1.5 统计分析

采用Cochrane协作网提供的RevMan 5.0进行*meta*分析。计数资料采用相对危险度(relative risk, RR)为疗效分析统计量; 计量资料采用加权均数差(WMD)或标准化均数差(SMD), 各效应量均以95%可信区间(CI)表示。各纳入研究结果间的异质性采用*Chi*^2^检验, 当各研究间有统计学同质性(*P* > 0.1, *I*^2^ < 50%)时, 采用固定效应模型对各研究进行*meta*分析; 如各研究间存在统计学异质性(*P* < 0.1, *I*^2^ > 50%), 分析其异质性来源, 对可能导致异质性的因素进行亚组分析, 若两个研究组之间存在统计学异质性而无临床异质性或差异无统计学意义时, 采用随机效应模型进行分析。异质性源于低质量研究, 进行敏感性分析。如两组间异质性过大或无法找寻数据来源时, 采用描述性分析。

## 2 结果

### 2.1 纳入研究数量

初检文献144篇, 阅读标题、摘要, 排除非随机对照试验、重复发表、非临床研究文献, 最终纳入14个研究。

### 2.2 纳入研究一般情况及质量评价(表 1)

**1:** 纳入研究一般情况和质量评价 Statistics and assessment quality of included studies

Trial	Paients (NO/NP)	Age (NO/NP)	Conslusion	Randomization	Allocated concealment	Blinding	Loss of follow-up
Feng YH^[4]^	25/25	62.50/63.30	ORR, DCR, AEs	UA	UA	UA	NMT
Gao JF^[5]^	58/32	63.50/63.00	ORR, DCR, 1-year survival, TTP, OS, AEs	Random number table	UA	UA	DS
Huang P^[6]^	25/23	73.00	ORR, DCR, AEs	UA	UA	UA	DS
Liu MZ^[7]^	40/42	60.00/57.00	ORR, DCR, AEs	UA	UA	UA	NMT
Liu ZT^[8]^	41/62	58.00/54.00	ORR, DCR, AEs	UA	UA	UA	NMT
Li XW^[9]^	25/25	58.00	ORR, DCR, AEs	Simple random sampling	UA	UA	NMT
Li YC^[10]^	26/24	68.00	ORR, DCR, AEs	UA	UA	UA	NMT
Lu J^[11]^	30/30	63.00/62.00	ORR, DCR, AEs	UA	UA	UA	NMT
Ren L^[12]^	83/85	56.00	ORR, DCR, 1-year survival rate, AEs	UA	UA	UA	NMT
Wang XH^[13]^	64/62	58.00/56.00	ORR, DCR, AEs	UA	UA	UA	NMT
Wu YM^[14]^	56/53	50.00	ORR, AEs	UA	UA	UA	NMT
Yao WX^[15]^	29/30	54.00	ORR, DCR, AEs	Simple random sampling	UA	UA	NMT
Zhang XR^[16]^	81/34	57.00/54.50	ORR, DCR, TTP, 1-year survival, AEs	Block Random	UA	UA	DS
Zhang GL^[17]^	34/36	57.00/56.00	ORR, DCR, 1-year survival, AEs	UA	UA	UA	NMT
ORR: objective response rate; DCR: disease control rate; AEs: adverse effects; TP: time to progression; UA: unclear; NMT: not mentioned; DS: described.							

14个研究均提到随机分组, 其中1个研究^[4]^采用随机数字表产生随机序号, 2个研究为单纯随机抽样^[9, 15]^, 1个研究为区组随机^[16]^。所有研究未报道分配隐藏和盲法, 3个研究^[5, 6, 16]^报道有失访, 但未进行ITT分析。

### 2.3 *meta*分析结果

#### 2.3.1 客观缓解率

14个研究^[4-17]^报告了客观缓解率, 各研究间无统计学异质性(*Chi*^2^=5.32, *P*=0.97, *I*^2^=0%), 采用固定效应模型, *meta*分析结果显示NO方案与NP方案的客观缓解率差异无统计学意义(RR=0.91, 95%CI:0.79-1.04)(图 1)。

**1:**
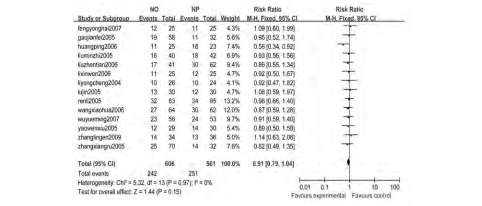
长春瑞滨联合奥沙利铂对比长春瑞滨联合顺铂治疗中晚期非小细胞肺癌的客观缓解率*meta*分析结果 The *meta*-analysis of overall response rates with NO and NP for advanced non-small cell lung cancer

#### 2.3.2 疾病控制率

13个研究^[4-13, 15-17]^报告了疾病控制率, 各研究间无统计学异质性(*Chi*^2^=5.76, *P*=0.89, *I*^2^=0%), 采用固定效应模型, *meta*分析结果显示NO方案与NP方案的疾病控制率差异无统计学意义(RR=0.98, 95%CI:0.92-1.04)(图 2)。

**2:**
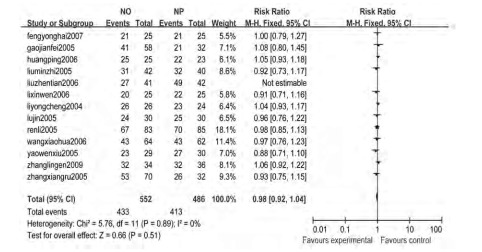
长春瑞滨联合奥沙利铂对比长春瑞滨联合顺铂治疗中晚期非小细胞肺癌的疾病控制率*meta*分析结果 The *meta*-analysis of disease control rates with NO and NP for advanced non-small cell lung cancer

#### 2.3.3 1年生存率

4个研究^[5, 12, 16, 17]^报道1年生存率, 各研究间无统计学异质性(*Chi*^2^=3.90, *P*=0.27, *I*^2^=23%), 采用固定效应模型, *meta*分析结果显示NO方案与NP方案的1年生存率差异无统计学意义(RR=0.96, 95%CI:0.76-1.23)(图 3)。

**3:**
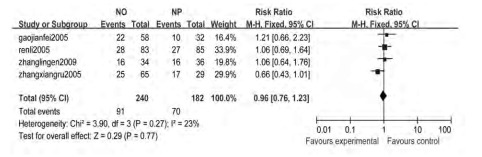
长春瑞滨联合奥沙利铂对比长春瑞滨联合顺铂治疗中晚期非小细胞肺癌的1年生存率*meta*分析结果 The *meta*-analysis of 1-year survival rate s with NO and NP for advanced non-small cell lung cancer

#### 2.3.4 毒副作用

##### 2.3.4.1 Ⅲ/Ⅳ度消化道毒性

14个研究^[4-17]^均报告Ⅲ/Ⅳ度消化道毒性, 各研究间无统计学异质性(*Chi*^2^=20.74, *P*=0.08, *I*^2^=41%), 采用固定效应模型, *meta*分析结果显示NP方案Ⅲ/Ⅳ度消化道毒性明显高于NO方案(RR=0.25, 95%CI:0.17-0.35, *Z*=8.02, *P* < 0.001)。

##### 2.3.4.2 Ⅲ/Ⅳ度血液学毒性

12个研究^[4-6, 9-17]^报告了Ⅲ/Ⅳ度白细胞减少, 各研究间无统计学异质性(*Chi*^2^=10.91, *P*=0.36, *I*^2^=8%), 采用固定效应模型, 结果显示NP方案Ⅲ/Ⅳ度白细胞减少高于NO方案, 具有统计学差异(RR=0.75, 95%CI:0.58-0.97, *Z*=2.16, *P*=0.03)。6个研究^[4, 10, 11, 13, 16, 17]^报道Ⅲ/Ⅳ度贫血, *meta*分析显示NO方案和NP方案差异无统计学意义(RR=0.77, 95%CI:0.37-1.58)。7个研究^[4, 11, 13-17]^报告Ⅲ/Ⅳ度血小板减少, 两方案差异无统计学意义(RR=0.96, 95%CI:0.59-1.55)。

##### 2.3.4.3 Ⅲ/Ⅳ度脱发

4个研究^[4, 9, 10, 15]^报告Ⅲ/Ⅳ度脱发, 各研究间无统计学异质性(*Chi*^2^=0.42, *P*=0.94, *I*^2^=0%), 采用固定效应模型, *meta*分析结果显示NP方案Ⅲ/Ⅳ度脱发发生率明显高于NO方案(RR=0.38, 95%CI:0.20-0.73, *Z*=2.92, *P*=0.004)。

##### 2.3.4.4 Ⅲ/Ⅳ度神经毒性

11个研究^[4, 5, 7, 8, 10-16]^报告了Ⅲ/Ⅳ度神经毒性, 各纳入研究间无统计学异质性(*Chi*^2^=0.98, *P*=0.99, *I*^2^=0%), 采用固定效应模型, *meta*分析结果显示NO方案Ⅲ/Ⅳ度神经毒性发生率明显高于NP方案(RR=3.75, 95%CI:1.48-9.48, *Z*=2.79, *P*=0.005)。

##### 2.3.4.5 Ⅰ/Ⅱ度肾毒性

由于Ⅲ/Ⅳ度肾毒性罕见, 故本研究仅评价Ⅰ/Ⅱ度肾毒性。纳入的12个研究^[4-6, 8, 10-17]^间无统计学异质性(*Chi*^2^=12.67, *P*=0.17, *I*^2^=29%), 采用固定效应模型, *meta*分析显示NP方案Ⅰ/Ⅱ度肾毒性明显高于NO方案(RR=0.46, 95%CI:0.32-0.68, *Z*=3.98, *P* < 0.0001)。

## 3 讨论

1995年, NSCLC协作组的*meta*分析确立了含铂方案在NSCLC治疗中的地位^[20]^, 第一代DDP与NVB联合的NP方案成为初治晚期NSCLC的标准方案^[21]^。十几年来, NP方案广泛应用, 疗效肯定, 但DDP的不良反应如肾毒性、消化道毒性使部分高龄及身体状况欠佳患者难以耐受, 限制了DDP的广泛应用。

随着第三代铂类OXA的问世, 以OXA为基础的联合方案能否具有更优异的疗效和安全性, 成为临床医生关心的课题。OXA通过烷化结合作用于DNA, 在碱基上形成链内和链外交联, 形成的复合体较顺铂产生的铂复合体大, 抑制DNA的合成与复制, 与顺铂相比具有水溶性高、细胞毒性强、肾毒性低和胃肠道反应小等优点。美国国立癌症研究所抗癌药物筛选中心针对DDP和草酸铂活性的研究发现, 两者的抗瘤谱不同, 体内和体外无交叉耐药性, 草酸铂可用于对顺铂耐药患者。NVB作为一种植物类细胞周期特异性抗癌药, 可抑制微管蛋白聚合、干扰微管蛋白的形成和诱导微管蛋白解聚, 使细胞分裂停止于有丝分裂中期。Depierre等^[22]^报道NVB单药治疗晚期NSCLC有效率为16%, 与顺铂或草酸铂联合治疗晚期NSCLC的有效率分别为43%和35%。

本研究通过*meta*分析对比OXA与NVB联合的NO方案与传统NP方案的疗效与副作用, 最终的结论与各项研究的结果基本一致:NO方案和NP方案在客观缓解率、疾病控制率、1年生存率方面的差异无统计学意义, 由于多数研究缺乏疾病进展时间及总生存期的数据, 结果仅提示两方案的近期疗效相似。副作用方面, NP方案的消化道毒性、肾毒性、白细胞减少、脱发发生率较NO方案增高, 差异有统计学意义(*P* < 0.05)。消化道毒性是由于DDP损伤消化道粘膜导致肠上皮嗜铬细胞释放5-羟色胺, 刺激迷走神经的5-羟色胺受体释放导致严重呕吐。肾毒性的发生主要由于DDP致氧化损伤、肾小管上皮细胞内钙超载及肾血管收缩、血流量、肾小球滤过率下降, 引起肾小管上皮细胞急性坏死、变性、肾间质水肿和肾小管扩张等, 其对肾小管的损害一般是可逆的, 但在大剂量或连续投药时, 可使肾小管损伤表现为不可逆性^[23]^。临床上DDP和水化、利尿通常同时进行, 老年及心肺功能不全患者很难耐受。NO方案表现出较高的神经毒性, 相比NP方案具有统计学差异(*P* < 0.05), 主要表现为周围神经炎, 症状可逆, 停药后可在一周内恢复, 与OXA在脊髓背根中心神经元的清除速度较慢, 并可生成草酸盐等物质损伤神经有关^[24]^。对于上述患者, NO方案可能更具优势。

本系统评价纳入的研究质量高低不等:①只有4个研究^[5, 9, 15, 16]^描述具体随机方法, 其它研究仅提及随机, 所有研究均未提及使用分配隐藏及盲法, 这可能导致选择偏倚、实施偏倚以及测量偏倚的可能性; 3个研究^[5, 6, 16]^提到失访, 但未进行ITT分析, 可能引起失访偏倚; ②部分测量指标如生存率等纳入研究较少或结果报道不充分, 使论证强度受到影响; ③所有纳入研究均来自国内, 这可能影响结果的普适性; ④部分测量指标和数据报道不充分, 如只有1个研究^[5]^报道了中位生存期, 2个研究^[5, 16]^虽提及疾病进展时间, 但是没有提供两组治疗前后的数据, 无法进行统计分析。今后随机对照研究应详细提供每一随访年的生存率结果, 进行更长期随访观察并报道终点指标的详细数据。此外, 采用随机、充分实施分配隐藏、实施双盲, 提高研究报告的质量; 还应对临床经济学、病人生活质量等方面进行报告, 以求更全面了解NO方案与NP方案在治疗中晚期NSCLC方面的优劣, 指导临床决策。

NO方案与NP方案治疗中晚期NSCLC疗效相似, 但副作用有差异, 我们倾向于认为NO方案与传统的NP方案相比, 疗效相似但耐受性好, 对于身体状况不佳的老年患者及不能耐受恶心呕吐、脱发、大量水化的患者, NO方案可能更适合作为治疗的首选方案。
